# AutoTemplate: enhancing chemical reaction datasets for machine learning applications in organic chemistry

**DOI:** 10.1186/s13321-024-00869-2

**Published:** 2024-06-27

**Authors:** Lung-Yi Chen, Yi-Pei Li

**Affiliations:** 1 Department of Chemical Engineering, National Taiwan University, No. 1, Sec. 4, Roosevelt Road, Taipei, 10617 Taiwan; 2 Taiwan International Graduate Program on Sustainable Chemical Science and Technology (TIGP-SCST), No. 128, Sec. 2, Academia Road, Taipei, 11529 Taiwan

**Keywords:** Reaction template, Data preprocessing, Atom-to-atom mapping, Reaction data curation

## Abstract

**Abstract:**

This paper presents AutoTemplate, an innovative data preprocessing protocol, addressing the crucial need for high-quality chemical reaction datasets in the realm of machine learning applications in organic chemistry. Recent advances in artificial intelligence have expanded the application of machine learning in chemistry, particularly in yield prediction, retrosynthesis, and reaction condition prediction. However, the effectiveness of these models hinges on the integrity of chemical reaction datasets, which are often plagued by inconsistencies like missing reactants, incorrect atom mappings, and outright erroneous reactions. AutoTemplate introduces a two-stage approach to refine these datasets. The first stage involves extracting meaningful reaction transformation rules and formulating generic reaction templates using a simplified SMARTS representation. This simplification broadens the applicability of templates across various chemical reactions. The second stage is template-guided reaction curation, where these templates are systematically applied to validate and correct the reaction data. This process effectively amends missing reactant information, rectifies atom-mapping errors, and eliminates incorrect data entries. A standout feature of AutoTemplate is its capability to concurrently identify and correct false chemical reactions. It operates on the premise that most reactions in datasets are accurate, using these as templates to guide the correction of flawed entries. The protocol demonstrates its efficacy across a range of chemical reactions, significantly enhancing dataset quality. This advancement provides a more robust foundation for developing reliable machine learning models in chemistry, thereby improving the accuracy of forward and retrosynthetic predictions. AutoTemplate marks a significant progression in the preprocessing of chemical reaction datasets, bridging a vital gap and facilitating more precise and efficient machine learning applications in organic synthesis.

**Scientific contribution:**

The proposed automated preprocessing tool for chemical reaction data aims to identify errors within chemical databases. Specifically, if the errors involve atom mapping or the absence of reactant types, corrections can be systematically applied using reaction templates, ultimately elevating the overall quality of the database.

**Graphical Abstract:**

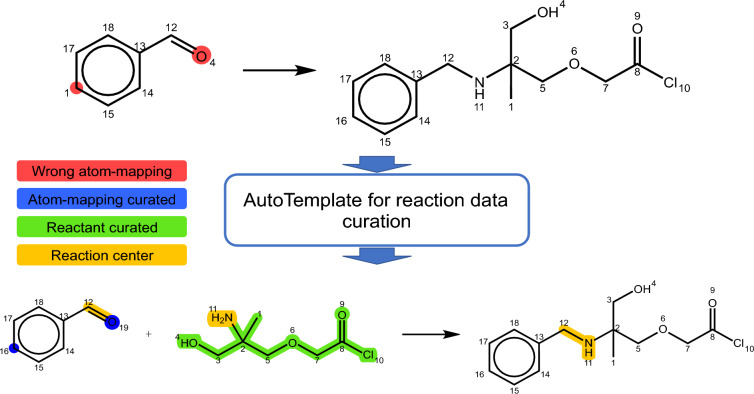

**Supplementary Information:**

The online version contains supplementary material available at 10.1186/s13321-024-00869-2.

## Introduction

Recent advancements in artificial intelligence have greatly expanded its applications in the field of chemistry. Machine learning techniques have been integrated into various aspects of organic synthesis, including yield prediction [1–4], forward prediction [5–10], retrosynthesis [11–23] and reaction condition prediction [24–27]. These predictive models rely on extensive and reliable chemical reaction datasets, enabling the development of robust machine learning solutions for real-world scenarios [28–32].

**Fig. 1:**
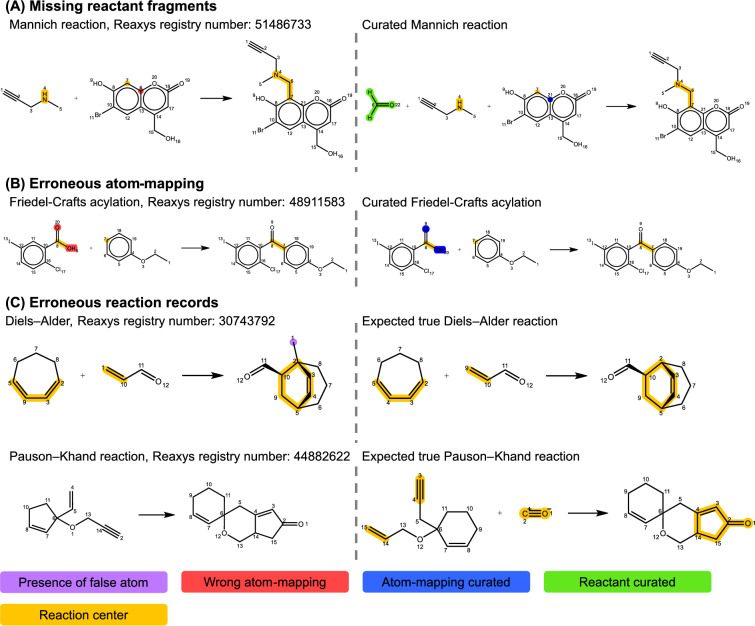
Common errors in chemical reaction datasets: **A** Missing reactant component; for instance, formaldehyde is omitted in the depicted Mannich reaction. **B** Incorrect atom mapping, either present in the dataset or generated by mapping software. **C** Two instances of erroneous reactions: the first displays a violation of the law of conservation of matter due to an unaccounted carbon atom (highlighted in purple), corrected on the right as per the study by De Nino et al. [49]; the second shows a mismatch between reactant and product, with the correct reaction displayed on the right, based on research by Özdemirhan [50]. These examples are sourced from the Reaxys database [36], but it is important to note that similar errors exist in other databases. Notably, the original Reaxys dataset lacks atom-mapping information, and the atom-mapping labels in the left half of this figure were generated using the RXNMapper software [43]

Chemical reaction databases commonly utilized in the literature can be broadly categorized as open-source datasets such as the United States Patent and Trademark Office (USPTO) [33] and open reaction database (ORD) [34], or proprietary datasets like Pistachio [35], Reaxys [36], SciFinder [37], and Spresi [38]. These datasets are compiled through text-mining or manual recording, both of which can introduce errors in the chemical reaction data. Figure 1 illustrates common data deficiencies observed in chemical databases, including missing reactants, inexplicable extra atoms in products, and even entirely erroneous reactions. Detecting and rectifying these data inconsistencies often require human intervention to ensure the quality of machine learning models.

To address these issues, Gimadiev et al. [39] employed atom-to-atom mapping toolkits [40–43] and the CGRTools [44] python library for preprocessing chemical transformations. They used a condensed graph of reaction (CGR), representing the superposition of the reactants and products, to remove duplicate reactions and balance reaction equations, particularly in cases where simple reagents like amine and water were unspecified. In contrast, Vaucher et al. [45] developed a transformer-based model [46] to complete reaction equations by filling in missing parts of molecules in partial reactions using a sequence-to-sequence approach. Although the model exhibited versatility in handling retrosynthesis, forward prediction, and data curation tasks, it achieved an accuracy of approximately 30% for exact matches, which may pose limitations in its application for extensive preprocessing of external chemical reaction datasets. More recently, Toniato et al. [47] employed the concept of catastrophic forgetting [48] to monitor the learning progress of molecular transformer [9] during training. Data points with difficulty in learning were assumed to be associated with errors and were subsequently removed from the dataset. However, the extent of data removal using this approach significantly depended on the model used, its learning capacity, and hyperparameter selection, rendering it less deterministic.

To the best of our knowledge, existing data-preprocessing methods have limited capacity to detect and correct false chemical reactions simultaneously. This gap has motivated us to develop an advanced data-preprocessing protocol called AutoTemplate in this work. AutoTemplate establishes clear criteria for identifying and removing erroneous data while effectively recovering missing reactants. It assumes that the majority of reactions in a dataset provide a reliable foundation for generating accurate templates. By employing these templates for data curation, AutoTemplate can successfully identify incorrect reactions, correct faulty atom mapping, and complete missing reactants, providing a solid foundation for the development of data-driven machine learning models, thereby enhancing the performance of forward and retrosynthetic predictions.

## Method

**Fig. 2:**
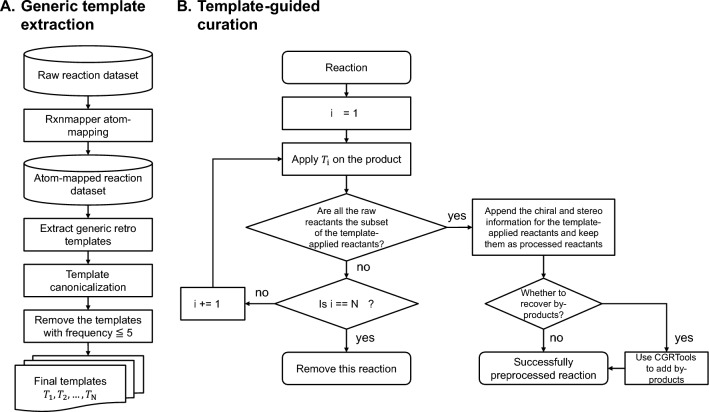
Overview of the two-stage data cleaning protocol of AutoTemplate for processing chemical reaction data. **A** illustrates the generic template extraction procedure. **B** shows the template-guided reaction curation process, which systematically validates the reaction data using a list of generic reaction templates

The data cleaning methodology presented in this work is divided into two stages: generic template extraction and template-guided reaction curation. In the generic template extraction stage, we first identify meaningful reaction transformation rules within the dataset of interest. These rules are then expressed as generic reaction templates using a simplified version of the SMARTS representation [51]. This simplification ensures that the templates can be applied to a wide range of reactions with the same transformation. In the template-guided reaction curation stage, we leverage the list of generic reaction templates to systematically validate the reaction data. This involves applying retro templates to the product. If the original reactants are indeed a subset of the results obtained through template application, the template-applied outcomes replace the original data. This process effectively rectifies any missing reactant information and simultaneously corrects potential atom-mapping errors. However, in situations where none of the templates match the reaction, indicating an unusual chemical transformation and potentially incorrect data entry, we opt to remove that specific reaction from our dataset. The overall procedure is visually depicted in Fig. 2, with detailed step-by-step explanations provided in the following subsections.

### Generic template extraction

#### Reaction data collection

To evaluate the effectiveness of our data cleaning protocol, we applied it to reaction data derived the Reaxys database [36], a well-established resource in the field of computational chemistry that, like any large database, may contain some errors [39]. To demonstrate the broad applicability of our data preprocessing approach, we retrieved datasets for 20 different reaction types from Reaxys. These datasets were obtained by searching for specific reaction names, and they encompassed a variety of reactions, including Adams decarboxylation, Baylis-Hillman reaction, Buchwald-Hartwig cross coupling, Chan-Lam coupling, Diels-Alder, Fischer indole synthesis, Friedel-Crafts acylation, Friedel-Crafts alkylation, Grignard reaction, Hiyama coupling, Huisgen cycloaddition, hydrogenation, Kabachnik-Fields reaction, Kumada coupling, Mannich reaction, Negishi coupling, Pauson-Khand reaction, reductive amination, Suzuki coupling, and Wittig reaction. The Reaxys registry number for each reaction used in our study are provided in the GitHub repository for reference [52]. We removed any reactions involving reactants or products that could not be parsed by RDKit [53]. In addition, we eliminated isotope labels from the molecules since they do not impact the chemical transformation. It is worth noting that the labels denoting reaction types in the Reaxys database may not always align accurately with the actual reaction types. Therefore, despite our efforts to collect data based on the 20 specified reaction names, there were instances where the recorded reaction entries did not correspond precisely to these 20 designated reaction types.

#### Atom-to-atom mapping

The original reaction data obtained from Reaxys lacked information on atom mapping, a crucial element for establishing correspondence between the atoms of reactants and products. This information is essential to identify the reaction center where the connectivity of atoms has changed, a prerequisite for extracting the reaction template. The accuracy of common atom-to-atom mapping toolkits has been assessed in the study by Lin et al. [41]. According to their findings, the open-source tool RXNMapper [43] demonstrated state-of-the-art performance, processing each reaction within one second. It is important to acknowledge that accurate atom mapping in chemical reactions often requires the reactions to be stoichiometrically balanced [54]. However, many entries in chemical databases do not fully comply with this requirement, presenting challenges in atom mapping. Currently, RXNMapper, which we use in our study, does not include functionality for stoichiometry correction [55], which can lead to unpaired atom mapping numbers when reaction SMILES lack balanced reactant and product entries. Further investigation into methodologies for enhancing atom mapping accuracy in such scenarios is necessary and could significantly advance the field.

With atom-mapping information available, we can distinguish spectator molecules—those that do not actively participate in the reaction or contribute any non-hydrogen atoms to the product. In our data preprocessing framework, spectator molecules are initially removed to concentrate on the core chemical transformations essential for effective template extraction. However, recognizing the importance of these molecules in various chemical contexts, such as yield prediction, we provide an option within our framework for users to reintroduce these initially removed spectator molecules post-data processing. This flexibility allows users to tailor the dataset to better fit their specific research needs, ensuring both clarity in template generation and comprehensiveness in reaction data.

#### Generic template definition and extraction

Upon obtaining the atom-mapped reactions, the next step is to retrieve all the reaction templates from the dataset using the RDChiral [56] template extractor. It is important to note that RDChiral primarily focuses on generating retrosynthetic templates, which are designed for developing computer-aided retrosynthesis models. By applying these templates to the products documented in the dataset, we can infer and reconstruct the reactants necessary to form these products. This process enables us to identify and supplement any missing reactants in the reaction entries, thereby enhancing the completeness and accuracy of our chemical reaction database.

The default templates generated by RDChiral provide highly detailed information around the reaction center. This results in an excessive number of templates for the same type of chemical transformation, particularly when there are minor variations in neighboring functional groups. It also extends the time required for the subsequent template application process. The specificity of these templates can make it challenging to apply a template from one reaction entry to curate another entry, unless both entries have identical neighboring functional groups near the reaction center. To overcome these challenges, we made modifications to the RDChiral functions. Our aim was to create generic reaction templates that include only essential information concerning atom types and bond types within the reaction centers, while excluding extraneous details. Table 1 provides a comparison between the default and modified template extraction functions.

**Fig. 3:**
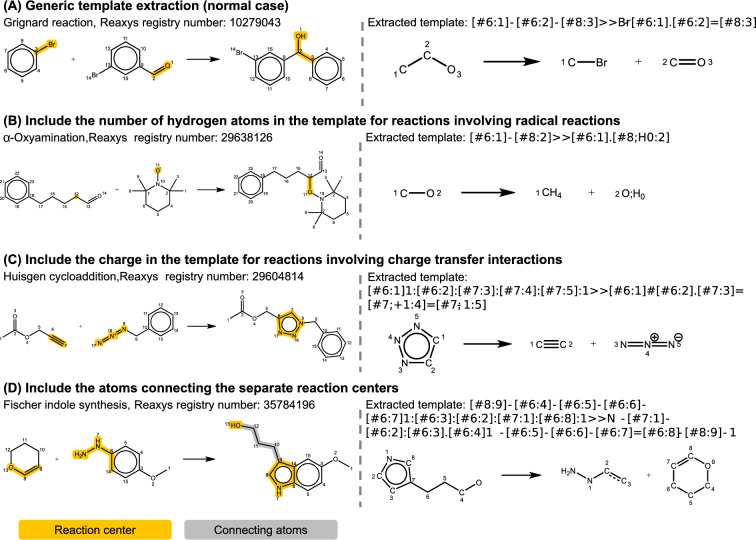
Illustration of generic template extraction with the normal and special cases

Consider the Grignard reaction in Fig. 3A as an example, the corresponding reaction template generated by default RDChiral is [OH;D1;+0:4]-[CH;D3;+0:5](-[c:6])-[c;H0;D3;+0:1](:[c:2]):[c:3]>>Br-[c;H0;D3;+0:1](:[c:2]):[c:3].[O;H0;D1;+0:4]=[CH;D2;+0:5]-[c:6]. On the other hand, its generic template reduces to [#6:1]-[#6:2]-[#8:3]>>Br-[#6:1].[#6:2]=[#8:3]. In the generic template, details related to atomic aromaticity, degree of freedom, number of hydrogen atoms, charge, and extra atoms are all discarded. The meanings of the notations used in the template can be found in the reaction SMARTS documentation [57]. This simplification effectively documents the chemical transformation for most cases. Nevertheless, there are special cases that require unique treatment. The first exception involves specifying the number of connected hydrogens in the generic template to accurately represent species involved in radical reactions, as shown in Fig. 3B. The second exception is the inclusion of the number of charges in the template when the reaction involves charge transfer, as illustrated in Fig. 3C. The third exceptional case arises when separate reaction centers occur in the product (Fig. 3D). In such cases, the connecting atoms between the reaction centers should be incorporated into the generic template. These connecting atoms can be identified using Dijkstra’s algorithm [58], which finds the shortest path between given nodes. This approach ensures that no redundant atoms are included in the template and is effectively applicable to extracting templates for ring-opening reactions.

**Table 1:** The features specified in default RDChiral and generic reaction templates

Level	Features	RDChiral	Generic
Atom	Reactant radius$^{1}$	1	0
	Product radius$^{1}$	0	0
	Aliphatic or aromatic	Yes	No
	Degree of freedom$^{2}$	Yes	No
	Chirality	Yes	No
	No. of hydrogen atoms	Yes	No, except for radical reactions
	Charge	Yes	No, except for charge transfer reactions
Bond	Bond type	Yes	Yes
	Cis-trans isomerism	Yes	No
Functional groups	Leaving groups	Yes	Yes
	Predefined groups	Yes	No

$^{1}$Radius denotes the extending distance of the neighbor atoms around the reaction center

$^{2}$Degree of freedom here represents the number of connecting non-hydrogen atoms

#### Template canonicalization

**Fig. 4:**
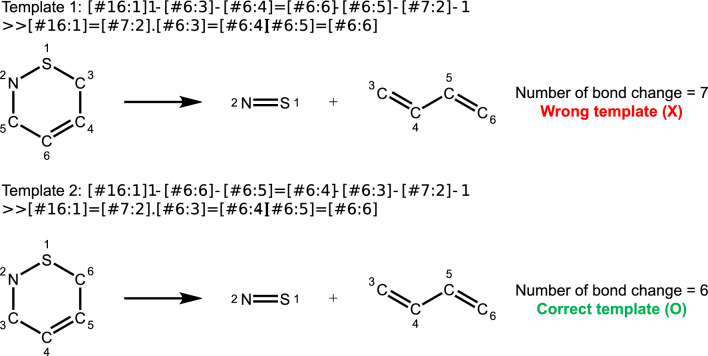
Examples of two generic templates extracted from Diels-Alder reactions

To address the issue of having multiple generic reaction templates representing the same chemical transformation but with different text representations [59], we employed a graph isomorphism check to confirm whether the reactants and products in pairwise templates were identical. If both reactant and product SMARTS patterns were graph isomorphic, we combined the two templates. Additionally, we calculated the number of bond changes in the templates and keep the one with fewer changes. Figure 4 illustrates this scenario with two Diels-Alder reaction templates that share identical subgraphs of reactants and products but differ in reaction transformations due to mapping errors from the atom-mapping tool. Such errors can lead to incorrect atom swaps, resulting in additional and incorrect formation and breaking of chemical bonds. Therefore, we retained the template with fewer bond changes. This concept drew inspiration from the principle of minimum chemical distance (PMCD) [60], a heuristic principle that assumes most chemical reactions follow the shortest path of bond change to convert reactants into products. Nevertheless, this assumption may fail in certain rare instances, such as in mechanistically complex reactions involving resonance-mediated bond transformations, as demonstrated by Chen et al. [61]. Clarifying exceptions within chemical reactions is deemed challenging, thus we leave it for future work.

#### Removal of rare templates

Generic templates are designed to be broadly applicable to reaction instances with similar chemical transformations. If a generic template matches only a few reaction entries, it suggests an unusual chemical transformation, possibly indicating that the template may have been derived from a reaction entry with errors. To address this, we monitored the occurrence frequency of each generic template during the template extraction process. Templates with a frequency of 5 or less were removed. This process resulted in the final set of generic templates $\left\{ T_1,T_2,\cdots T_N\right\}$ for subsequent template-guided curation.

### Template-guided curation

#### Template application procedure

This procedure primarily involves the iterative application of generic reaction templates to the products of each reaction entry. When the reactants in the original data entry form a subset of the reactants resulting from the applied template, we replace the original data’s reactants with those from the applied template. This rectifies any missing reactant information and simultaneously corrects potential atom-mapping errors. In cases where none of the templates match the reaction, indicating an unusual chemical transformation and potentially incorrect data entry, we choose to remove that specific reaction entry from the dataset.

Throughout the template application process, the reactants are automatically supplemented with the appropriate number of hydrogen atoms based on their charge state and the number of bonds connected to them. For instance, neutral sulfur atoms are assigned either two or six bonds, resulting in two possible configurations for a neutral sulfur atom with a connected chemical bond, acquiring either one or five hydrogen atoms. Exceptions to this rule only occur when the template explicitly specifies the number of hydrogen atoms connected to the reaction center.

#### Append atomic chirality and bond stereochemistry

We note that the reactants generated from template application lack annotations for atomic chirality and bond stereochemistry at the reaction centers. Therefore, an additional step is necessary to reintroduce this information into the reactants, but only if this information was included in the original dataset. This process involves establishing a one-to-one atom correspondence between the original reactants and template-generated reactants. This can be achieved by initially converting both sets of reactants into undirected graphs, followed by utilizing the exact graph matching algorithm [62] to establish a strict one-to-one node correspondence between the two graphs.

#### Reaction balance with addition of by-products

Chemical reaction datasets commonly record only the primary products, frequently omitting by-products such as those derived from leaving groups. To address this gap, tools like CGRTools have been employed to augment atom-mapped reaction SMILES by integrating hydrogen atoms into leaving groups, thus representing them as electroneutral by-products [44]. This functionality has been incorporated into our software, allowing users to optionally implement this step during data processing. Figure S4 showcases a reaction that has been modified to include by-products. It is crucial to recognize, however, that this method is a simplification and may not fully capture the complexities of by-product formation in actual chemical reactions. Therefore, the analysis and discussions in this paper do not extend to the detailed curation of by-products.

## Results and discussion

### Analysis of overall results

Table 2 provides information on the number of reactions in the dataset, the number of templates extracted from these reactions, and the residual proportion after data processing. The residual proportion is calculated as the percentage of chemical reactions that successfully undergo template-guided curation and remain in the dataset, relative to the total number of reactions initially present. The variation in the number of templates for each type of reaction is due to the unique characteristics of their reaction mechanisms. For example, coupling reactions that involve multiple possible leaving groups often result in a higher template count. Conversely, reductive amination, where the carbonyl group is reduced to an amine, has a large number of reaction entries, but only 16 reaction templates are extracted, indicating less variation in its reaction transformation.

**Fig. 5:**
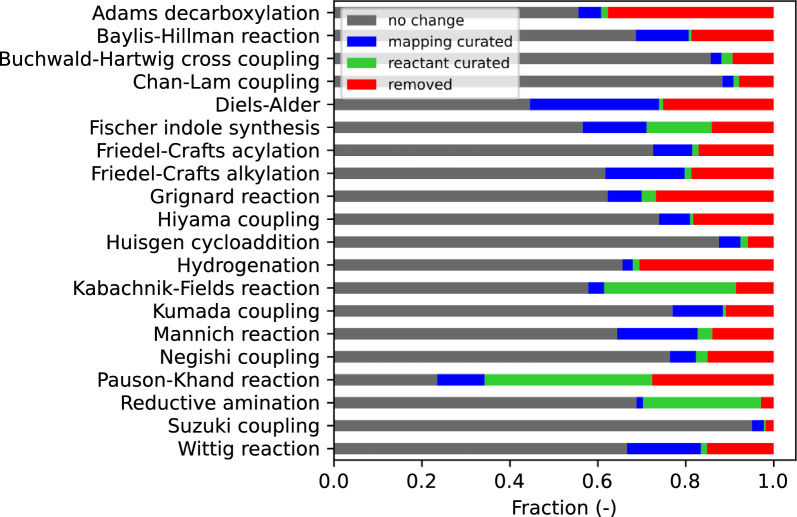
Distribution of the proportion of repaired reactions after data processing

Figure 5 displays curated reaction results, addressing issues such as false atom-mapping, reactant omissions, and the identification and removal of incorrect reaction records. To assess the proportion of curation for reactant omissions, we compare the number of reactant molecules before and after data processing; an increase indicates that missing reactants have been successfully added to the reaction formula. For the curation proportion concerning atom-mapping, we evaluate the consistency of CGR representations of reactions before and after processing, and any discrepancies suggest modifications in the atom-mapping. We acknowledge that the methodologies adopted for automating large-scale analysis of these processed datasets may not perfectly delineate the success of data cleansing. However, based on our empirical observations, these methodologies are sufficiently effective. Notably, the Diels-Alder reactions exhibited a high atom-mapping correction rate of 29.3%. This is likely attributed to the complexity of Diels-Alder reactions, which involve numerous bond transformations and instances of intramolecular or fused ring formation, making them challenging for accurate atom-mapping predictions. Conversely, coupling reactions generally showed relatively fewer atom-mapping errors, likely because they involve fewer bond changes. Accurate atom-mapping data can significantly improve reaction prediction quality, particularly for graph-based models. Regarding the issue of missing reactants, Fischer indole synthesis, Kabachnik-Fields reaction, Pauson-Khand reaction, and reductive amination display a noteworthy proportion of data with absent reactants. In the case of the Pauson-Khand reaction, most instances systematically omit carbon monoxide as a reactant. However, there is no clear pattern indicating which reactants may be omitted in the data for the other three types of reactions. Further discussions on specific data errors and curated results are provided in the following subsections for selected examples.

**Table 2:** The data preprocessing results for the chemical reaction datasets

Reaction type	No. of reactions	No. of generic templates	Residual proportion (%)
Adams decarboxylation	2636	54	62.3
Baylis-Hillman reaction	7507	84	81.3
Buchwald-Hartwig cross coupling	18,341	96	90.7
Chan-Lam coupling	6885	43	92.1
Diels-Alder	18,757	258	74.8
Fischer indole synthesis	6841	28	85.9
Friedel-Crafts acylation	10,095	118	82.9
Friedel-Crafts alkylation	17,248	164	81.3
Grignard reaction	13,530	154	73.2
Hiyama coupling	4089	106	81.7
Huisgen cycloaddition	54,183	144	94.1
Hydrogenation	41,217	306	69.4
Kabachnik-Fields reaction	5575	14	91.4
Kumada coupling	16,371	82	89.1
Mannich reaction	29,698	271	86.0
Negishi coupling	10,909	146	84.9
Pauson-Khand reaction	2703	19	72.4
Reductive amination	50,406	16	97.1
Suzuki coupling	184,219	216	98.2
Wittig reaction	16,337	94	84.8

### Examples of correcting atom-mapping errors

**Fig. 6:**
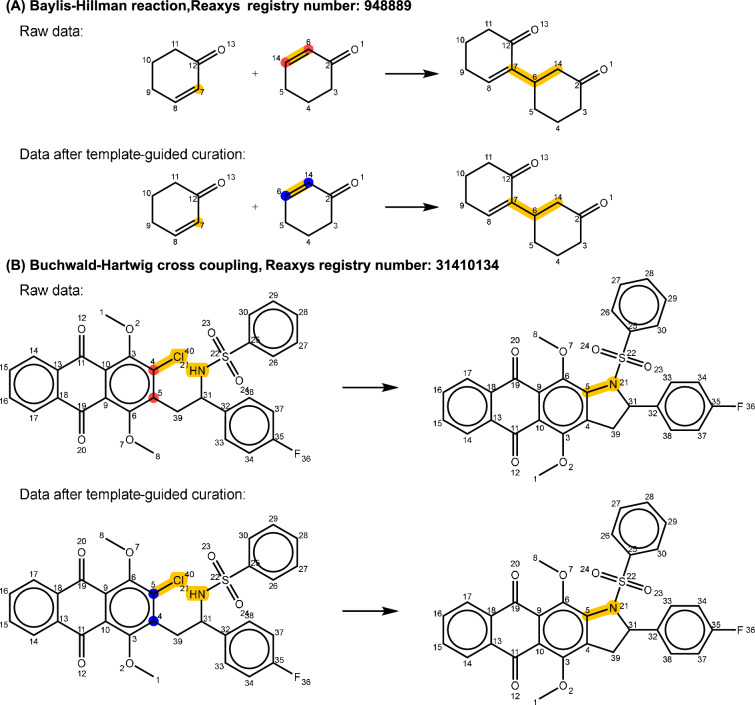
Two selected examples of **A** Baylis-Hillman reaction and **B** Buchwald-Hartwig cross coupling to demonstrate the curated results of the reaction entries with incorrect atom-mapping. Yellow highlights indicate the reaction centers, red highlights denote atoms with incorrect atom mapping, and blue highlights represent atoms with curated mapping

Currently, there is no package available that can generate atom-mapping information perfectly for all reactions [41]. In this study, the data-driven neural network RXNMapper [43] was utilized to predict atom mapping. However, it is important to note that even for reactions considered relatively straightforward for humans, there can still be instances of incorrect atom mapping, as shown in Fig. 6A. This example of the Baylis-Hillman reaction incorrectly assigns the atom-mapping number (6 and 14) at the position of the carbon-carbon double bond. This error results in a reaction template that displays more bond changes than templates derived from correctly mapped reactions of the same type. During our template canonicalization process, if the reactant and product SMARTS patterns in two templates are graph isomorphic, we merge them and retain only the one with fewer bond changes. As a result, the incorrect template is discarded, and the correct template, derived from other accurately mapped Baylis-Hillman reactions, is used to correct the atom mapping. Another example is the Buchwald-Hartwig cross-coupling reaction illustrated in Fig. 6B, which has the same issue at the reaction center where the carbon atoms are labeled incorrectly in the intramolecular ring-closing reaction. We note that false atom-mapping issues occur more frequently at the reaction centers. Systematically resolving these inaccuracies remains a significant challenge for atom-mapping generation tools. Addressing this problem would substantially benefit downstream applications that rely on template-based and graph-based modeling techniques.

### Examples of addressing missing reactant errors

**Fig. 7:**
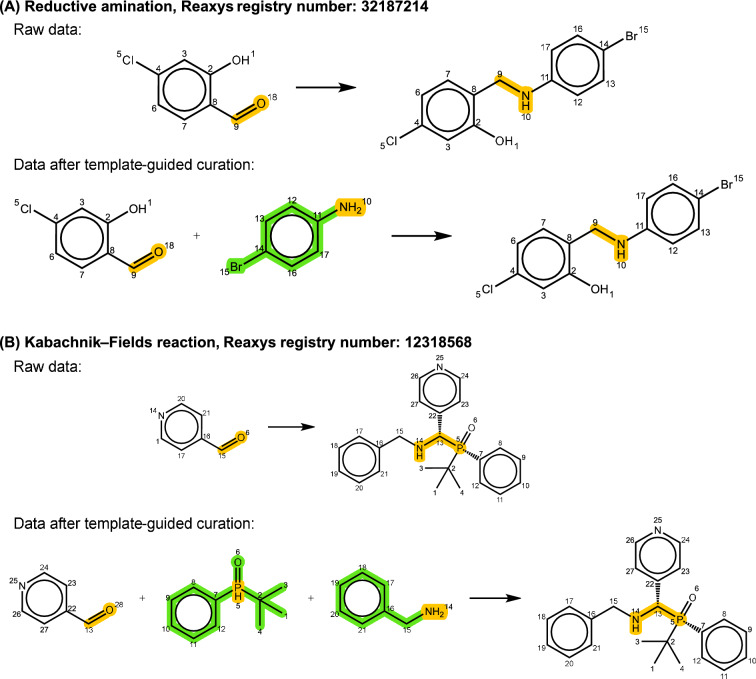
Two selected examples of **A** reductive amination and **B** Kabachnik-Fields reaction to demonstrate the curated results of the reaction entries with incomplete reactant information. Yellow highlights represent the reaction centers, while green highlights indicate molecular fragments added through the data curation process

The issue of missing reactants can be identified by comparing the atom counts between reactants and products, with reactions having fewer atoms on the reactant side categorized as this type of error. To the best of our knowledge, there is no existing approach tailored for adding missing reactants. However, with the template-guided curation method proposed in this work, erroneous reaction entries can be recovered along with the omitted reactants. Figure 7A illustrates a typical example from the reductive amination dataset, where the missing reactant with an amine functional group was generated by applying the generic template to the product, thus balancing the reaction equation. In the case of the second instance of the Kabachnik-Fields reaction shown in Fig. 7B, which involves three molecules in the reaction, the two missing fragments were successfully recovered with the help of template. It is worth noting that the chirality of the phosphorus atom cannot be inferred because the generic template does not specify chiral and cis-trans stereoisomerism at the reaction center. Including such detailed information in templates would lead to an excessive number of templates, reducing the chances of applying a template from one reaction entry to curate another entry.

### Examples of identifying and resolving erroneous reactions

**Fig. 8:**
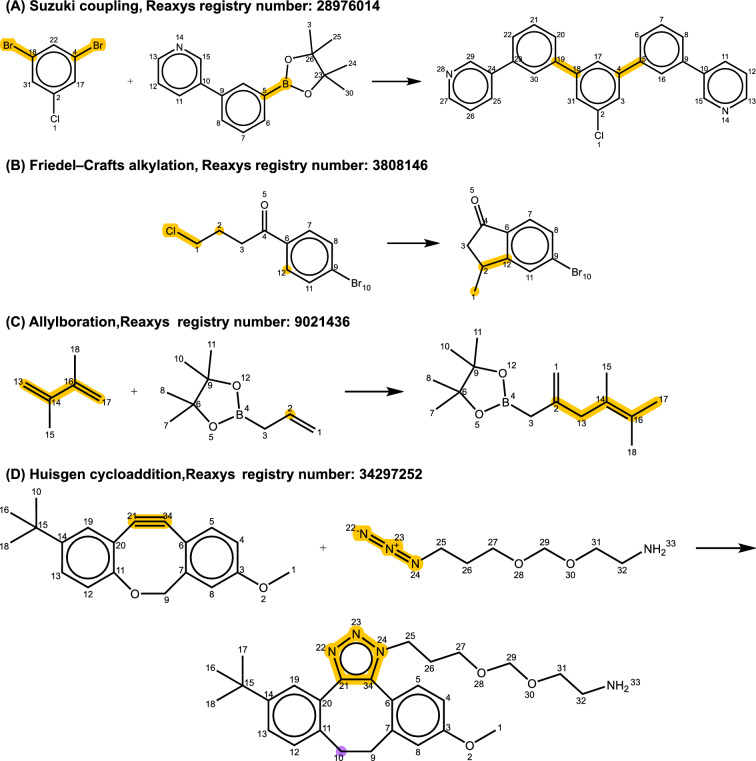
Four selected examples of **A** Suzuki coupling, **B** Friedel-Crafts alkylation, **C** allylboration, and **D** Huisgen cycloaddition to illustrate reactions that did not match any of the final generic templates and were consequently removed during the data processing procedure

In cases where none of the templates matched the reaction, indicating an unusual chemical transformation or potential data entry errors, the specific reaction entry was removed from the dataset. Several examples of such removals are presented in Fig. 8 and discussed below.

Figure 8A illustrates a two-step Suzuki coupling reaction. To automatically identify multi-step reactions like this, one would need to repetitively validate them using all the single-step reaction templates, which becomes increasingly time-consuming as the number of steps allowed grows. Because most reaction prediction models focus on single-step reactions, the accommodation of multi-step reactions is less critical in this study. The reactions shown in Fig. 8B and C are actually correct reactions, but none of the generic templates in the final list match them. This occurred because the templates derived from these reactions did not meet the minimum frequency threshold required for inclusion. As discussed in the method section, templates with low matching frequencies may indicate errors in the template source data. While this approach effectively removes erroneous reaction entries, it can also inadvertently exclude rare but valid reactions, as demonstrated in Fig. 8B and C. The reaction depicted in Fig. 8D belongs to the category of Huisgen cycloaddition. In this reaction, the atom highlighted in purple (number 10) in the product is identified as a carbon atom. However, at the same position in the reactant, an oxygen atom is indicated. Rectifying this type of error is challenging because it is difficult to determine whether the correct structure should be attributed to the reactant or the product. This particular entry originates from a study by McNitt et al. [63], where atom number 10 was labeled as an oxygen atom, suggesting a potential error in the recorded product information in the database.

### Evaluating the efficacy of template-guided curation under simulated error conditions in the USPTO-50k dataset

The above analysis details the results from applying our template-guided curation process to actual data records derived from a chemical database. However, comprehensively validating these curated results against the original chemical reactions poses a significant challenge due to the necessity of reviewing all source papers. To evaluate the efficacy of our template-guided curation in detecting and correcting errors, we deliberately introduced errors into the USPTO-50k dataset [12, 64], which already contains atom-mapping information and had been previously curated by Coley et al. [12].

To simulate potential errors typically found in real-world chemical databases, we introduced three types of noise into the USPTO-50k dataset: (1) removal of reactants, (2) structural modifications to products, and (3) swapping of atom mappings in products. The structural modifications included the addition of a carbon or oxygen atom, and the substitution of a carbon atom with either an oxygen or nitrogen atom. Each reaction in the dataset was altered to exhibit only one type of error at a time, with these three types of errors added with equal probability.

**Fig. 9:**
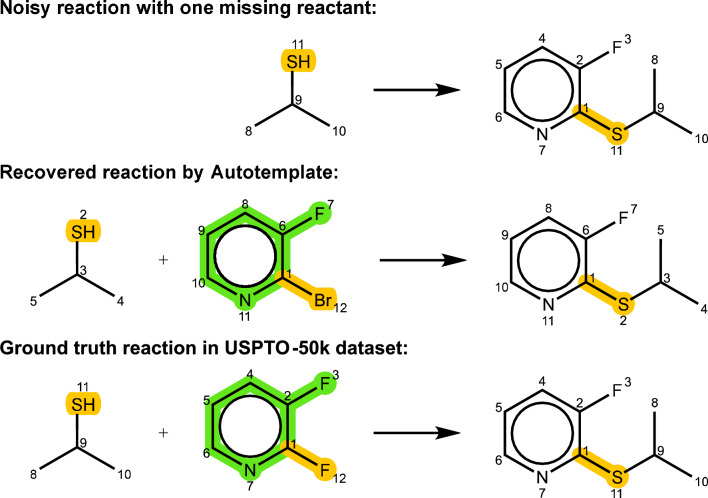
Impact of template selection on reactant structure. This figure shows how AutoTemplate selected a bromine template over a fluorine atom due to its higher frequency, leading to a change in the reactant structure. The USPTO patent number of this reaction is US05612288

We prepared datasets with varying error levels, resulting in total noise ratios of 9%, 15%, 30%, 45%, and 60%. Our template-guided curation was then applied to evaluate the efficacy of our method in handling each type of error. We assessed the success rates for accurately curating missing reactants and atom mappings, as well as the success rate for identifying and removing structurally modified erroneous reactions. Table 3 shows that despite varying levels of induced errors, our template-guided curation consistently detected and addressed different error types, demonstrating its robustness. Detection rates for erroneous reactions with structural changes were approximately 99%, and curation rates for atom-mapping errors were about 97%. However, the success rate for curating missing reactants was only around 62%. This lower rate is often due to challenges in restoring reactants when the missing reactant involves a leaving group not present on the product side. This issue is exacerbated when multiple leaving groups are applicable for a reaction, leading to the selection of different templates and resultant reactants. An example is shown in Fig. 9. In the original data, a fluorine atom is attached to carbon atom 1 in the product, but AutoTemplate selected a template attaching a bromine atom instead due to its higher occurrence frequency, resulting in a different reactant structure.

The USPTO-50k dataset’s diversity of leaving groups, including silicon-containing, and sulfonate, and other groups, intensifies the issue of competing templates, as shown in Fig. S5. This highlights a limitation in our template-guided curation process, making it challenging to restore the exact original reactants by merely selecting the most frequent template. Further study is needed to address this issue effectively. However, for the development of forward prediction models, these curated data with different leaving groups from the original data could still be valuable. As suggested by Wu et al. [65], virtual reaction data augmentation techniques that replace halogen atoms in certain coupling reactions can enhance the initial data volume and improve the accuracy of forward prediction models.

**Table 3:** Success rates (%) for handling different types of errors at various noise ratios

Error type	9% noise ratio	15% noise ratio	30% noise ratio	45% noise ratio	60% noise ratio
Missing reactants	62.00	62.08	61.49	62.24	61.36
Structurally modified erroneous reactions	99.67	99.40	99.64	99.67	99.70
Atom mapping errors	97.53	97.20	97.42	96.93	96.54

## Conclusions

Recent advancements in artificial intelligence have significantly impacted the field of organic chemistry. The reliability of predictive models in chemistry, essential for applications such as yield prediction, retrosynthesis, and reaction condition prediction, is heavily contingent on the quality of chemical reaction datasets. However, these datasets, sourced from both open-source and proprietary databases, sometimes contain inconsistencies like missing reactants, incorrect atom mappings, or erroneous reactions, necessitating rigorous data preprocessing.

This work introduces a novel data preprocessing protocol called AutoTemplate, designed to enhance the quality of chemical reaction datasets. AutoTemplate employs a two-stage approach: generic template extraction and template-guided reaction curation. The process begins with the extraction of meaningful reaction transformation rules from a dataset, which are then expressed as generic reaction templates using a simplified version of the SMARTS representation. This simplification ensures broad applicability across various reactions. In the subsequent stage, these generic templates are systematically applied to validate and correct reaction data. This involves rectifying missing reactant information, correcting atom-mapping errors, and removing incorrect data entries.

Our method is distinguished by its ability to identify and correct erroneous chemical reactions using simplified SMARTS templates derived from the dataset. This approach is based on the assumption that a majority of reactions provide a reliable basis for generating broadly applicable and accurate templates. By utilizing these templates for data curation, our AutoTemplate system not only addresses existing errors but also assists in restoring missing reactants. The protocol’s effectiveness is demonstrated through its application to diverse chemical reactions, highlighting significant improvements in dataset quality. This refined data offers a potentially more reliable foundation for developing machine learning models in chemistry, which could enhance the accuracy of forward and retrosynthetic predictions.

This study represents a significant step forward in preprocessing chemical reaction datasets, addressing a critical gap in the field and paving the way for more accurate and efficient machine learning applications in organic synthesis.

### Supplementary Information

Supplementary Material 1.

## Data Availability

Full code and Reaxys registry number for searching the reactions are available at: https://github.com/Lung-Yi/AutoTemplate.

## Abbreviations

CGR: Condensed Graph of Reaction
ORD: Open Reaction Database
PMCD: Principle of Minimum Chemical Distance
SMILES: Simplified Molecular Input Line Entry Specification
SMARTS: SMILES Arbitrary Target Specification
USPTO: United States Patent and Trademark Office
