# Supplementary material for: AutoTemplate: enhancing chemical reaction datasets for machine learning applications in organic chemistry
Source: J Cheminform. 2024 Jun 27;16:74. doi: 10.1186/s13321-024-00869-2 (PMC11212196; doi:10.1186/s13321-024-00869-2)
Supplement: Supplementary file 1 — Supplementary Material 1. [file 13321_2024_869_MOESM1_ESM.docx]

**AutoTemplate: Enhancing Chemical Reaction Datasets for Machine Learning Applications in Organic Chemistry**

Lung-Yi Chen^[a]^ and Yi-Pei Li^*[a][b]^

[a] Department of Chemical Engineering, National Taiwan University, No. 1, Sec. 4, Roosevelt Road, Taipei, 10617, Taiwan.

[b] Taiwan International Graduate Program on Sustainable Chemical Science and Technology (TIGP-SCST), No. 128, Sec. 2, Academia Road, Taipei, 11529, Taiwan.

*E-mail: [yipeili@ntu.edu.tw](mailto:yipeili@ntu.edu.tw)

Supporting Information

**Data Acquisition**

The reaction data sourced from Reaxys utilized in this study is derived from a proprietary database, necessitating subscription authorization for access. Consequently, we can only provide the Reaxys Registry Number for each reaction dataset as a means to facilitate the retrieval and reproduction of these data. These identifiers are available within this repository: <https://github.com/Lung-Yi/AutoTemplate/tree/main/data_reaxys>. The JSON files within the repository serve as import files for the Reaxys query builder; uploading and loading these JSON files into the following website enables direct searching of the data utilized in this study: <https://www.reaxys.com/#/search/advanced>. Subsequently, exporting the search results into XLSX files facilitates compatibility with the AutoTemplate code for data preprocessing.

Additionally, the USPTO-50k dataset employed in this study was extracted from the research conducted by Coley et al.,^1^ with the data source available at this URL: <https://raw.githubusercontent.com/connorcoley/retrosim/master/retrosim/data/data_processed.csv>.

Finally, Figure S1 illustrates the differences in the Reaxys chemical reaction data before and after data template-guided curation, as well as the number of extracted generic templates, which serve as a supplement to Table 2 in the manuscript.


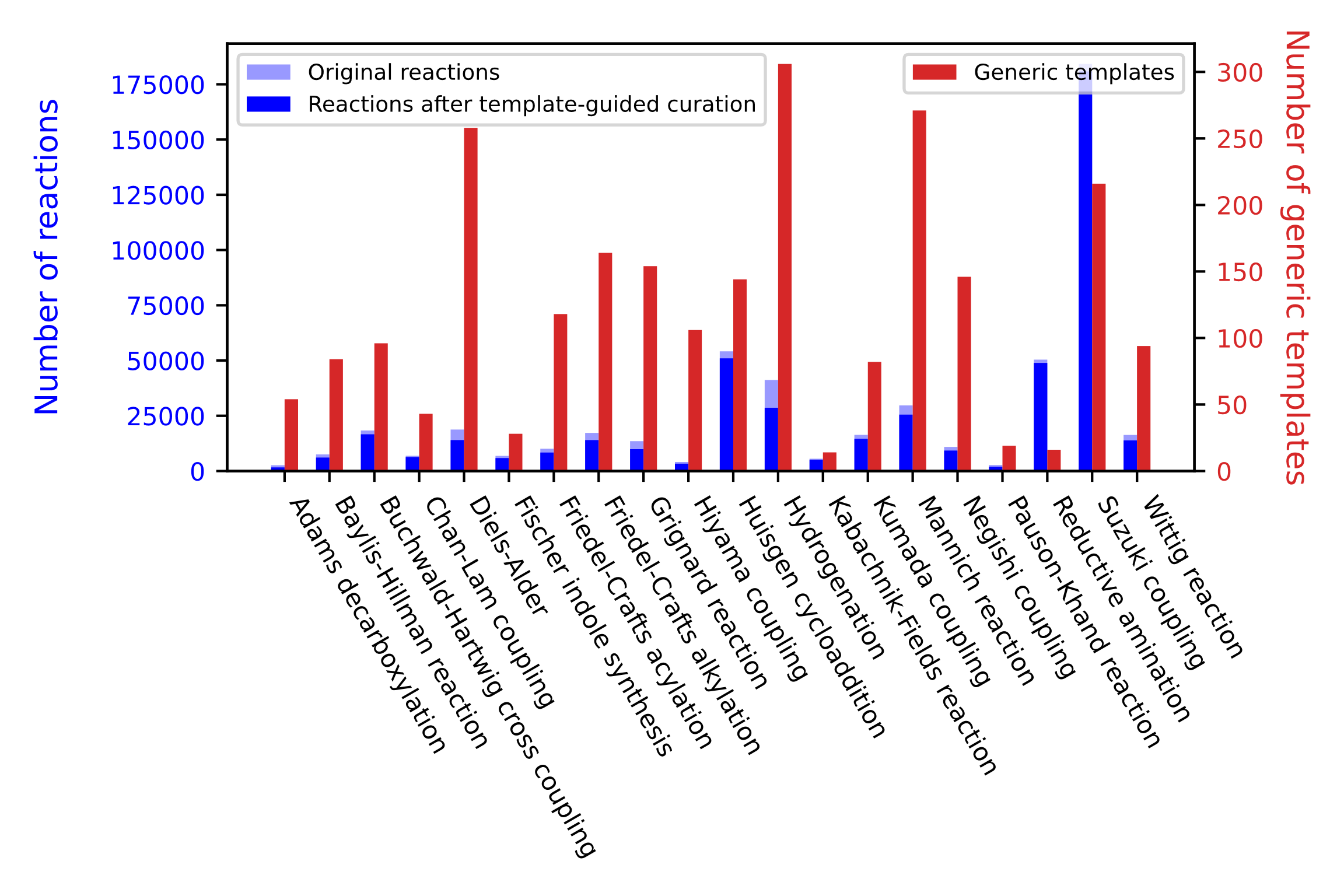


**Figure S1.** Data number before and after template-guided curation for each reaction and the number of extracted generic templates.

**Analysis of Generic and Aromaticity-Informed Templates**

The initial stage of our template-guided curation framework involves the extraction of meaningful reaction transformation rules and the formulation of generic reaction templates using a simplified SMARTS representation that excludes details such as atomic aromaticity, degree of freedom, number of hydrogen atoms, charge, and extraneous atoms. However, we have also explored the inclusion of aromatic/aliphatic distinctions in our templates, referred to as aromaticity-informed templates. In these templates, uppercase elemental symbols are used to denote aliphatic atoms, while lowercase symbols indicate aromatic atoms. For example, in a Suzuki coupling reaction (Figure S2), the generic reaction template is represented as "[#6:1]-[#6:2]>>Br-[#6:1].O-B(-O)-[#6:2]", contrasting with the aromaticity-informed template, which is represented as "[c:1]-[c:2]>>Br-[c:1].O-B(-O)-[c:2]".

To assess the impact of incorporating aromaticity information into reaction templates, we conducted an analysis comparing the preprocessing results using generic and aromaticity-informed templates on the Reaxys datasets, as detailed in Table S1. Our findings indicate that aromaticity-informed templates result in the extraction of a greater number of reaction templates due to the need to differentiate between aromatic and non-aromatic atoms. However, this specificity also leads to a reduction in the frequency of each template. For instance, the variability in template distribution for the Negishi coupling reaction family, which can occur on both aliphatic and aromatic carbon atoms, is illustrated in Figure S3. The most commonly occurring template in the generic form generates four distinct variations in the aromaticity-informed version, leading to a long-tailed distribution of template frequencies. In our data processing workflow, we remove templates appearing no more than five times to minimize the propagation of potentially erroneous reaction data. Consequently, although including the aromaticity information enhances the specificity of the templates used, it also leads to the exclusion of some correct templates that do not meet the frequency threshold. This selection criterion affects the ability to curate certain chemical reactions, resulting in a slight reduction in the number of residual reactions when using aromaticity-informed templates, compared to generic templates (Table S1).


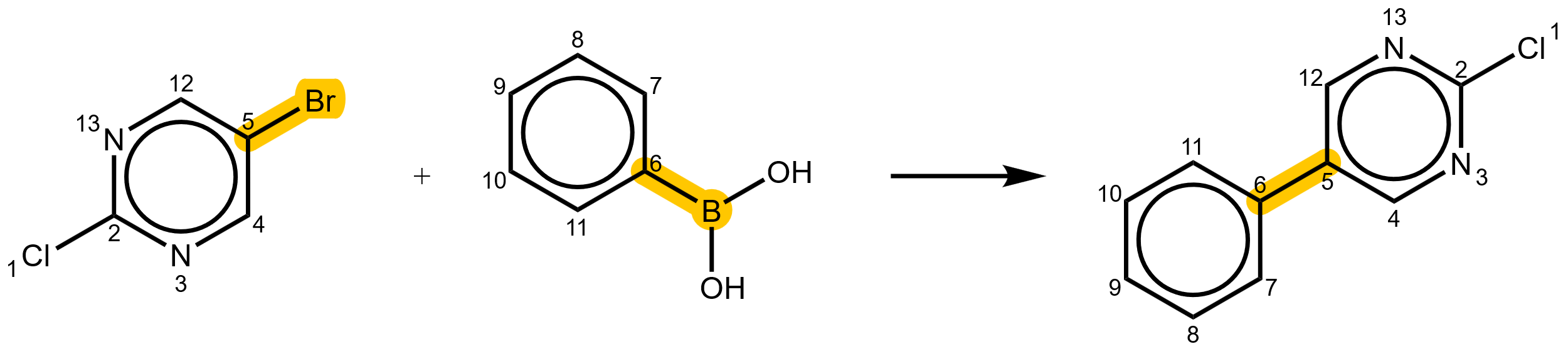


**Figure S2.** An example of Suzuki coupling reaction with its corresponding Reaxys registry number: 23053890. The atom-mapping labels in this figure were generated using the RXNMapper software.^2^ Yellow highlights indicate the reaction centers.

**Table S1.** Comparison of data curation results between generic and aromaticity-informed templates.

|  | Generic templates | | Aromaticity-informed templates | |
| --- | --- | --- | --- | --- |
| Reaction type | No. of templates | No. of residual reactions | No. of templates | No. of residual reactions |
| Adams decarboxylation | 54 | 1641 | 55 | 1595 |
| Baylis–Hillman reaction | 84 | 6101 | 87 | 6080 |
| Buchwald–Hartwig cross coupling | 96 | 16627 | 104 | 16545 |
| Chan–Lam coupling | 43 | 6342 | 52 | 6292 |
| Diels–Alder | 258 | 14032 | 266 | 14013 |
| Fischer indole synthesis | 28 | 5875 | 28 | 5867 |
| Friedel–Crafts acylation | 118 | 8368 | 121 | 8348 |
| Friedel–Crafts alkylation | 164 | 14017 | 170 | 13984 |
| Grignard reaction | 154 | 9903 | 186 | 9621 |
| Hiyama coupling | 106 | 3341 | 121 | 3192 |
| Huisgen cycloaddition | 144 | 50994 | 145 | 50982 |
| Hydrogenation | 306 | 28623 | 313 | 28574 |
| Kabachnik–Fields reaction | 14 | 5098 | 15 | 5098 |
| Kumada coupling | 82 | 14589 | 116 | 14397 |
| Mannich reaction | 271 | 25540 | 292 | 25447 |
| Negishi coupling | 146 | 9264 | 226 | 9071 |
| Pauson–Khand reaction | 19 | 1956 | 19 | 1956 |
| Reductive amination | 16 | 48931 | 16 | 48931 |
| Suzuki coupling | 216 | 180819 | 282 | 180536 |
| Wittig reaction | 94 | 13852 | 92 | 13833 |


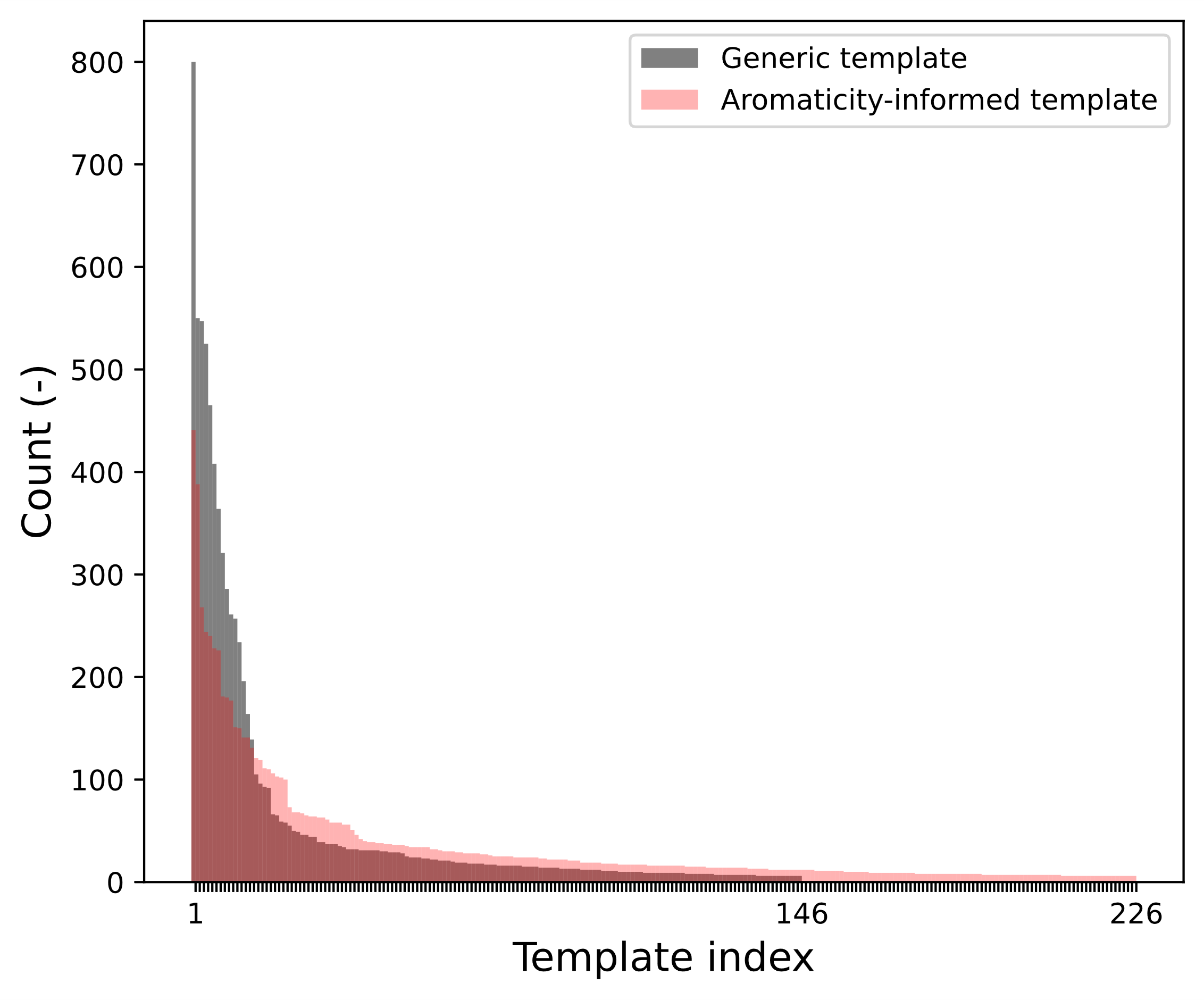


**Figure S3.** Distribution of generic and aromaticity-informed template frequencies for the Negishi coupling reaction. This figure shows only those templates that exceed the established frequency threshold.

**The Impact of Dataset Size on Data Curation Outcomes**

In chemical databases, the continuous influx of new data causes the dataset of chemical reactions to evolve dynamically. Given this variability, we explored how changes in dataset size impact the effectiveness of our template-guided data curation method. To assess this, we systematically reduced the Reaxys dataset into subsets representing 10%, 25%, 50%, and 75% of the total volume, applying the same processing procedures to each subset. Our findings, detailed in Table S2, demonstrate a clear correlation between the volume of data and the number of templates extracted for each reaction type.

As expected, smaller datasets feature less diversity in reactions and often do not meet the frequency threshold of five occurrences for certain templates, resulting in their exclusion. Consequently, using a smaller dataset yields a less comprehensive set of templates, which diminishes the proportion of chemical reactions that can be effectively curated. The efficacy of the curation process stabilizes only as the size of the dataset increases. To mitigate these limitations, using a pre-established template library could be beneficial, especially when curating new and smaller datasets. This approach leverages the reliability of templates extracted from a large, high-quality dataset to enhance the curation performance of smaller datasets.

**Table S2.** The data preprocessing results for down-sampled reactions. GT represents “generic template”, and RP (%) represents “residual proportion.”

|  | 10% | | 25% | | 50% | | 75% | | 100% | |
| --- | --- | --- | --- | --- | --- | --- | --- | --- | --- | --- |
| Reaction type | No. of GTs | RP | No. of GTs | RP | No. of GTs | RP | No. of GTs | RP | No. of GTs | RP |
| Adams decarboxylation | 5 | 37.26 | 9 | 42.79 | 25 | 53.79 | 40 | 59.18 | 54 | 62.25 |
| Baylis–Hillman reaction | 6 | 62.8 | 28 | 70.47 | 58 | 77.4 | 77 | 79.75 | 84 | 81.27 |
| Buchwald–Hartwig cross coupling | 14 | 84.62 | 29 | 86.37 | 56 | 89.04 | 77 | 90.19 | 96 | 90.65 |
| Chan–Lam coupling | 7 | 84.3 | 14 | 88.44 | 22 | 89.89 | 33 | 91.28 | 43 | 92.11 |
| Diels–Alder | 24 | 57.44 | 65 | 63.87 | 128 | 69.4 | 206 | 73.09 | 258 | 74.81 |
| Fischer indole synthesis | 4 | 82.46 | 10 | 83.68 | 18 | 84.62 | 22 | 85.28 | 28 | 85.88 |
| Friedel–Crafts acylation | 15 | 63.73 | 40 | 74.32 | 70 | 78.66 | 98 | 81.38 | 118 | 82.89 |
| Friedel–Crafts alkylation | 23 | 67.69 | 54 | 73.89 | 104 | 78.05 | 138 | 80.17 | 164 | 81.27 |
| Grignard reaction | 21 | 58.61 | 56 | 65.49 | 90 | 68.65 | 124 | 71.46 | 154 | 73.19 |
| Hiyama coupling | 15 | 46.08 | 40 | 63.7 | 68 | 74.51 | 93 | 79.58 | 106 | 81.71 |
| Huisgen cycloaddition | 15 | 89.07 | 42 | 91.37 | 91 | 92.60 | 121 | 93.00 | 144 | 94.11 |
| Hydrogenation | 43 | 57.68 | 83 | 62.45 | 162 | 65.72 | 226 | 67.61 | 306 | 69.44 |
| Kabachnik–Fields reaction | 3 | 86.71 | 10 | 90.45 | 11 | 90.53 | 12 | 90.74 | 14 | 91.44 |
| Kumada coupling | 13 | 83.02 | 29 | 85.61 | 49 | 87.42 | 67 | 88.52 | 82 | 89.11 |
| Mannich reaction | 41 | 72.79 | 80 | 77.33 | 167 | 82.68 | 218 | 84.68 | 271 | 86.00 |
| Negishi coupling | 28 | 65.69 | 61 | 76.16 | 107 | 82.53 | 131 | 84.05 | 146 | 84.92 |
| Pauson–Khand reaction | 3 | 65.56 | 5 | 66.52 | 11 | 69.21 | 14 | 70.45 | 19 | 72.36 |
| Reductive amination | 3 | 96.73 | 7 | 96.85 | 8 | 97.1 | 13 | 97.03 | 16 | 97.07 |
| Suzuki coupling | 31 | 96.57 | 74 | 97.36 | 122 | 97.81 | 165 | 98.02 | 216 | 98.15 |
| Wittig reaction | 12 | 75.93 | 26 | 79.14 | 50 | 81.71 | 77 | 84.07 | 94 | 84.79 |

**Recovery of By-Products**

Figure S4 illustrates an example of post-processing where by-products have been supplemented. The data processing function, "balance_rxn_smiles," used for this purpose is available at: <https://github.com/Lung-Yi/AutoTemplate/blob/main/autotemplate/graph_utils/reaction_balancing.py>


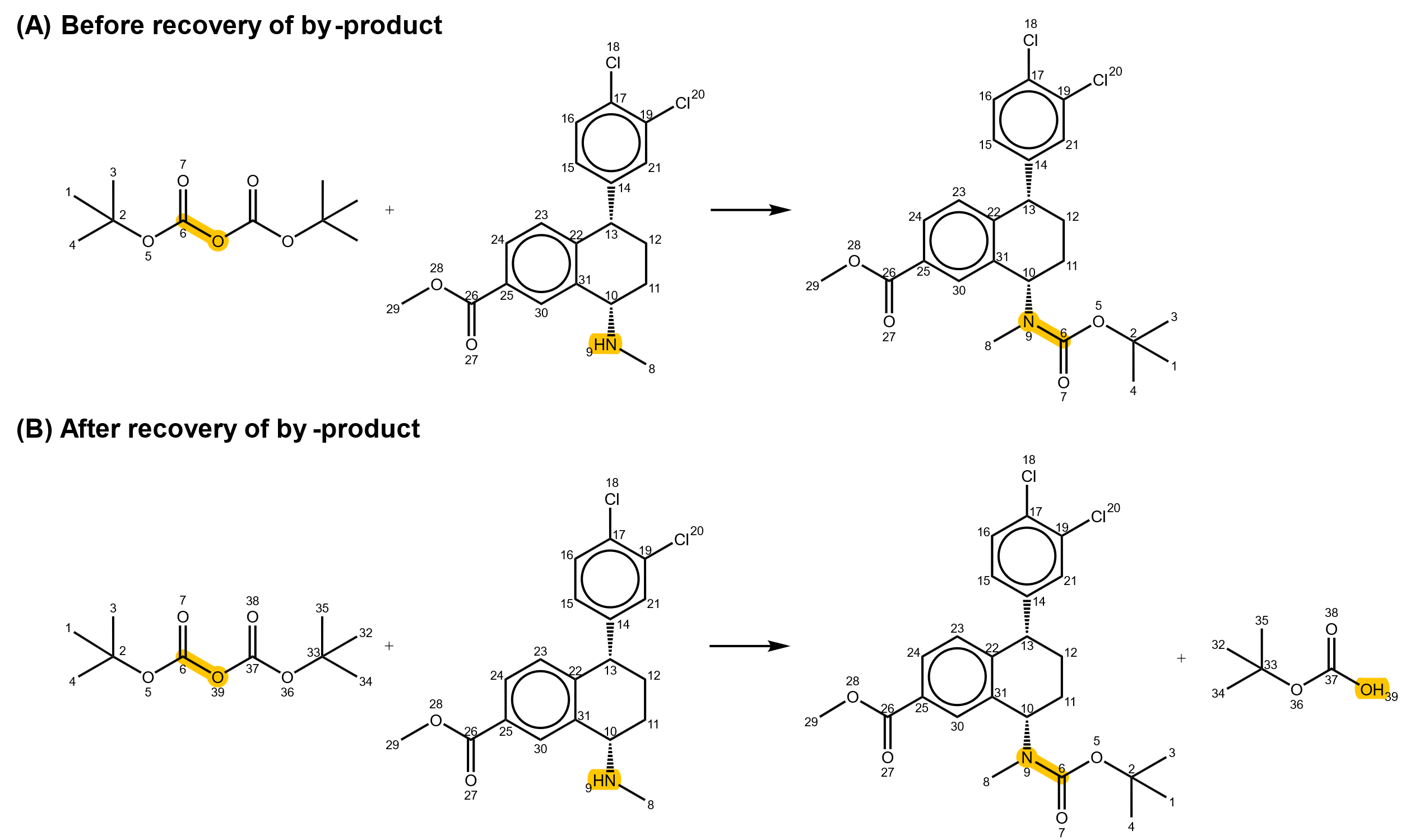


**Figure S4.** An illustrative example of by-product recovery using CGRTools.^3^ This reaction is taken from USPTO-50k dataset, and its corresponding USPTO patent number is US20100009970A1. Yellow highlights indicate the reaction centers.

**Challenges of Competing Templates in Restoring Missing Reactants**

Within the USPTO-50k dataset, numerous chemical reactions involve the release of various leaving groups to form products. Consequently, the templates extracted from these reactions often show identical product SMARTS but differ in their reactant SMARTS. This variety leads to what we term 'competing templates,' which, when used to address missing reactants, can result in the generation of different reactants associated with distinct leaving groups. Our analysis reveals that reactions with missing reactant errors are associated with an average of approximately 10 competing templates, as demonstrated in Figure S5. However, some reactions are linked to as many as 35 different templates, resulting in a broad range of potential reactant structures.

In our template-guided data curation process, we opt for the template with the highest occurrence frequency to generate the missing reactants. Although this approach effectively resolves many errors, it does not consistently replicate the precise original reactant structure for every reaction. This highlights the challenges and limitations of relying on frequency-based template selection in restoring accurate reactant structures.


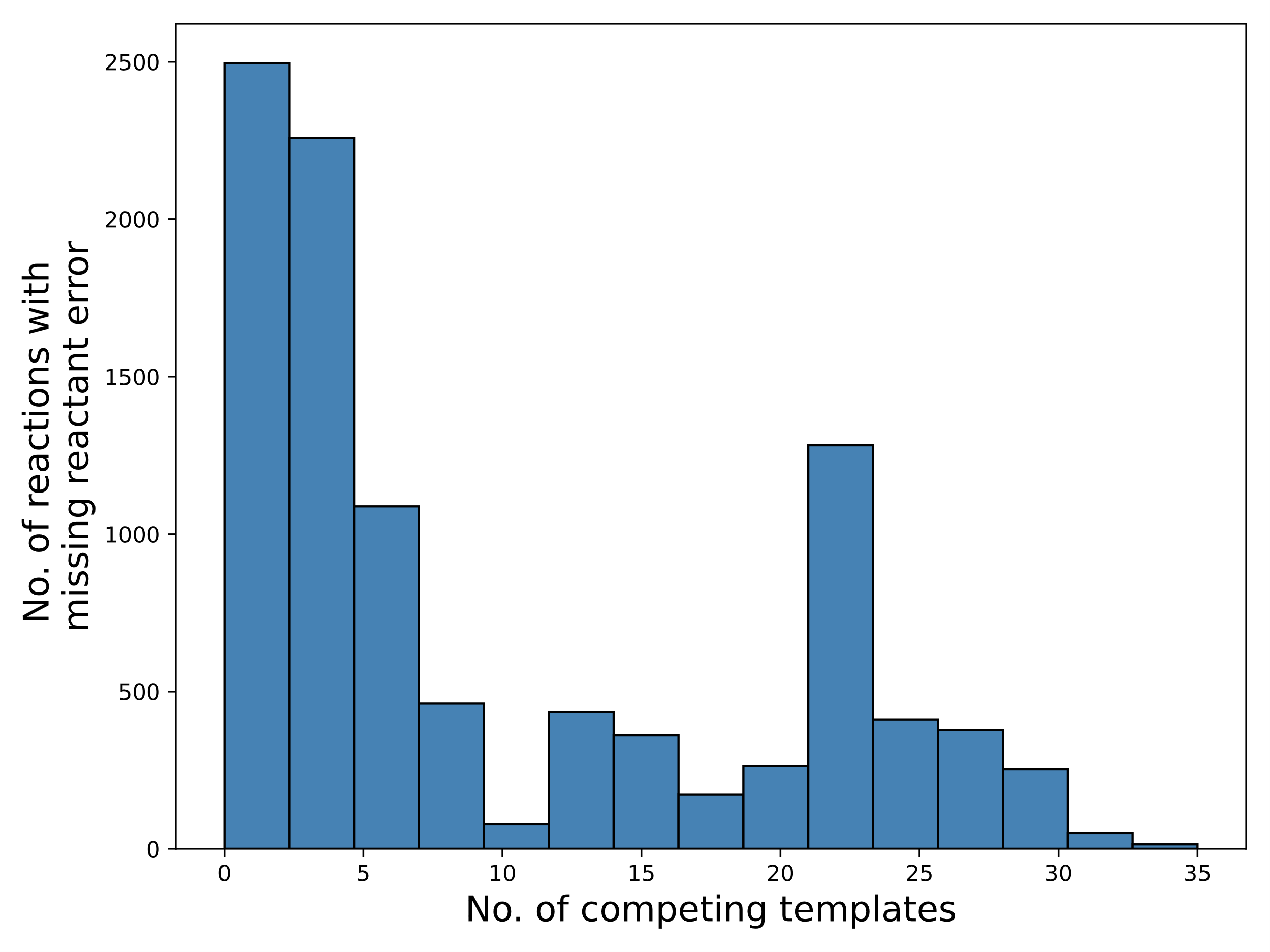


**Figure S5.** Distribution of competing templates. This figure illustrates the distribution of competing templates when 60% of the USPTO-50k dataset is artificially introduced with noise.

**References**

(1) Coley, C. W.; Rogers, L.; Green, W. H.; Jensen, K. F. Computer-assisted retrosynthesis based on molecular similarity. *ACS central science* **2017**, *3* (12), 1237-1245.

(2) Schwaller, P.; Hoover, B.; Reymond, J.-L.; Strobelt, H.; Laino, T. Extraction of organic chemistry grammar from unsupervised learning of chemical reactions. *Science Advances* **2021**, *7* (15), eabe4166.

(3) Nugmanov, R. I.; Mukhametgaleev, R. N.; Akhmetshin, T.; Gimadiev, T. R.; Afonina, V. A.; Madzhidov, T. I.; Varnek, A. CGRtools: Python library for molecule, reaction, and condensed graph of reaction processing. *Journal of chemical information and modeling* **2019**, *59* (6), 2516-2521.
